# Rituximab, etoposide, methylprednisolone, high-dose cytarabine, and cisplatin in the treatment of secondary hemophagocytic lymphohistiocytosis with classical Hodgkin lymphoma: a case report and review of the literature

**DOI:** 10.1186/s13256-016-1143-9

**Published:** 2016-12-20

**Authors:** Steve Hu, Pranshu Bansal, David Lynch, Cristhiam Mauricio Rojas Hernandez, Zoneddy Dayao

**Affiliations:** 1 Department of Internal Medicine, University of New Mexico, MSC10 5550, 1 University of New Mexico, 87131 Albuquerque, NM USA; 2 University of New Mexico Comprehensive Cancer Center, 1201 Camino de Salud NE, Room 3618, 87131 Albuquerque, NM USA; 3 Department of Pathology, University of New Mexico, MSC08 4640, 1 University of New Mexico, 87131 Albuquerque, NM USA; 4 University of Texas MD Anderson Cancer Center, 1515 Holcombe Blvd, Unit 1464, 77030 Houston, TX USA

**Keywords:** Hemophagocytic lymphohistiocytosis, Epstein–Barr virus, Hodgkin lymphoma, ESHAP, Rituximab, Pancytopenia, EBER, Case report

## Abstract

**Background:**

Hemophagocytic lymphohistiocytosis is becoming an increasingly recognized disorder in adults. Classical Hodgkin lymphoma is a relatively uncommon etiology of hemophagocytic lymphohistiocytosis and may complicate treatment options. Rituximab, etoposide, methylprednisolone, high-dose cytarabine, and cisplatin are discussed here as a treatment regimen.

**Case presentation:**

A 66-year-old Hispanic man previously in good health presented with a 1-month history of recurrent fevers, chills, and night sweats and a 3-week history of new onset jaundice. A bone marrow biopsy revealed a normocellular bone marrow with increased histiocytes with areas of hemophagocytic activity. He met five out of eight criteria for hemophagocytic lymphohistiocytosis diagnosis including fevers, pancytopenia, hemophagocytosis, ferritin of 23,292 ng/mL (>500 ng/mL), and soluble-CD25 of 15,330 pg/mL (>1033 pg/mL). A right cervical lymph node biopsy revealed CD15, CD30, MUM-1, and Epstein–Barr virus-encoded small ribonucleic acid-positive cells with morphologic findings of classical Hodgkin lymphoma, lymphocyte-rich subtype. He completed 2 weeks of hemophagocytic lymphohistiocytosis-directed therapy with etoposide and dexamethasone, but then was switched to rituximab, etoposide, methylprednisolone, high-dose cytarabine, and cisplatin due to minimal improvement in his pancytopenia and hepatic impairment. He completed one full cycle of rituximab, etoposide, methylprednisolone, high-dose cytarabine, and cisplatin with notable improvement in serial hepatic function panels and had an undetectable Epstein–Barr virus viral load. However, he eventually died due to complications of *Enterococcus faecalis* bacteremia and colonic microperforation in the setting of persistent pancytopenia.

**Conclusions:**

This case discusses the challenges facing treatment of adult malignancy-associated hemophagocytic lymphohistiocytosis. Rituximab, etoposide, methylprednisolone, high-dose cytarabine, and cisplatin may be a viable option for patients with secondary hemophagocytic lymphohistiocytosis and Hodgkin lymphoma who cannot tolerate standard therapies due to hepatic impairment. Targeted therapy and immunotherapy are promising new areas of developing treatments.

## Background

Hemophagocytic lymphohistiocytosis (HLH) is a rare life-threatening disorder that is often difficult to diagnose in the adult population. Characterized by a diffuse inflammatory state with a wide variety of clinical presentations, HLH is usually a delayed diagnosis [1]. This leads to an unfortunate delay in therapy in a disease with a rapid course and high mortality. To further complicate matters, current guidelines stem from the HLH-2004 protocol, which was derived from trials involving pediatric patients [2].

In contrast to pediatric HLH, adult HLH is more commonly secondary in nature and often therapy directed at treating HLH alone is insufficient. This is quite notable in the cases of malignancy-driven HLH. To complicate matters further, the progression of HLH itself can lead to hepatic dysfunction, making clinicians hesitate to use cytotoxic chemotherapy directed towards underlying malignancies. In these cases, the initial use of HLH-directed therapy in the form of dose-reduced etoposide and dexamethasone alone may not lead to effective recovery.

We discuss a case of secondary HLH with Epstein–Barr virus (EBV)-positive classical Hodgkin lymphoma. Hodgkin lymphoma can be reported in up to 10% of malignancy-associated HLH cases and there is a paucity of data to support an optimal chemotherapy regimen, especially in light of the dismal survival rates [3, 4]. We describe an attempt at a novel treatment in the form of rituximab, etoposide, methylprednisolone, high-dose cytarabine, and cisplatin (R-ESHAP), a therapy with less hepatotoxicity, in hopes of targeting the underlying EBV-positive Hodgkin lymphoma.

## Case presentation

A previously healthy 66-year-old Hispanic man presented with a 1-month history of recurrent fevers, chills, and night sweats and a 3-week history of new onset jaundice. His pertinent medical history included localized prostate cancer status post-transurethral resection of prostate in remission, peptic ulcer disease, benign prostatic hypertrophy, hypertension, hypothyroidism, and prior left lower extremity deep vein thrombosis several years ago. On physical examination, he was fatigued, febrile, tachycardic, with diffuse jaundice and mild lower extremity bilateral pitting edema. Just 6 months prior at a primary care physician visit, he had grossly normal laboratory values including complete blood count and liver function tests. A complete blood count on initial workup at our institute was significant for white blood cell count of 2.7×10^3^/μL, platelets of 98×10^9^/L, and hemoglobin of 8.2 g/dL with a mean corpuscular volume (MCV) of 78 fL. He had normal electrolytes, but abnormal liver function panel with a total protein of 5.2 g/dL, albumin of 1.3 g/dL, aspartate aminotransferase (AST) of 135 units/L, alanine aminotransferase (ALT) of 269 units/L, alkaline phosphatase of 1235 units/L, total bilirubin of 4.9 mg/dL, direct bilirubin 4.1 mg/dL, and indirect bilirubin 0.8 mg/dL. His international normalized ratio (INR) was elevated at 1.59. Blood cultures and urine analysis were unremarkable; a QuantiFERON Gold test was indeterminate. Further workup for acute liver failure with abdominal ultrasound, magnetic resonance cholangiopancreatography (MRCP), and hepatobiliary scan revealed possible findings of chronic cholecystitis but no biliary obstruction. He was empirically treated with ceftriaxone for assumed cholecystitis, but the results of his liver function panel tests continued to worsen. 

With his liver function panel and condition continuing to worsen, a more extensive autoimmune and infectious workup for liver failure was performed. A viral hepatitis panel revealed hepatitis A IgM, hepatitis B surface antigen and core IgM, and hepatitis C antibody to be negative. Further viral workup did show EBV IgG, cytomegalovirus (CMV) IgG, and herpes simplex virus (HSV) I IgG positivity. His EBV viral load was 55,000 copies/mL. EBV IgM, CMV IgM, human herpes virus-8 (HHV-8), human immunodeficiency virus (HIV), parvovirus B-19 IgG/IgM, and HSV I/II IgM were all negative. An autoimmune workup revealed negative antinuclear antibody (ANA), rheumatoid factor (RF), antiliver/kidney microsomal antibody, antimitochondrial antibody (AMA), and anti-smooth muscle antibody (ASMA). His ceruloplasmin and alpha-1 antitrypsin levels were normal, and he had a negative human hemochromatosis gene screening.

**Fig. 1:**
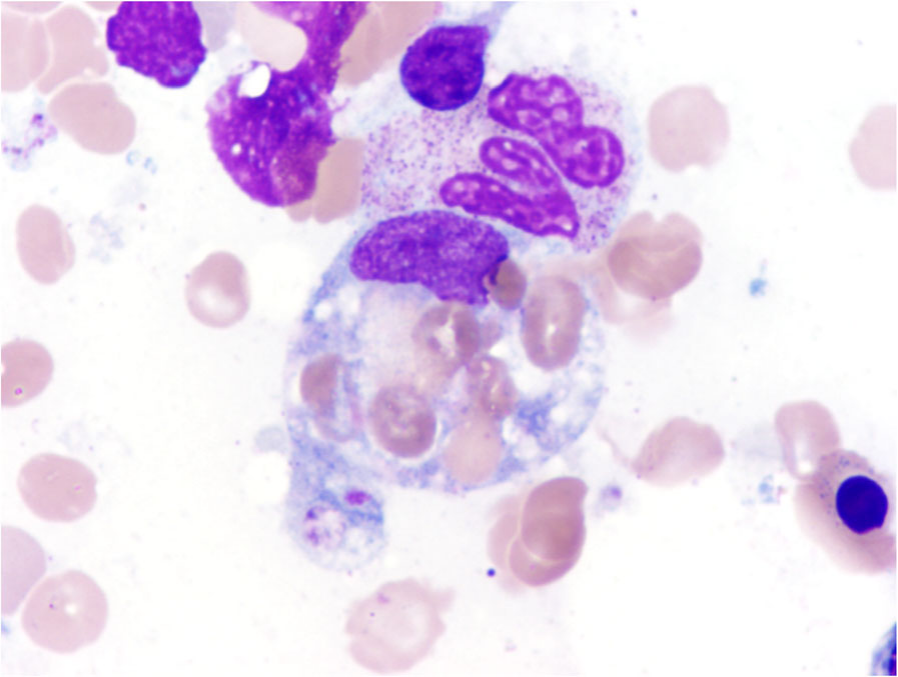
Hemophagocytic activity was present as evidenced by scattered histiocytes containing abundant red blood cells on the bone marrow aspirate. Wright-Giemsa, 1000× oil immersion

**Fig. 2:**
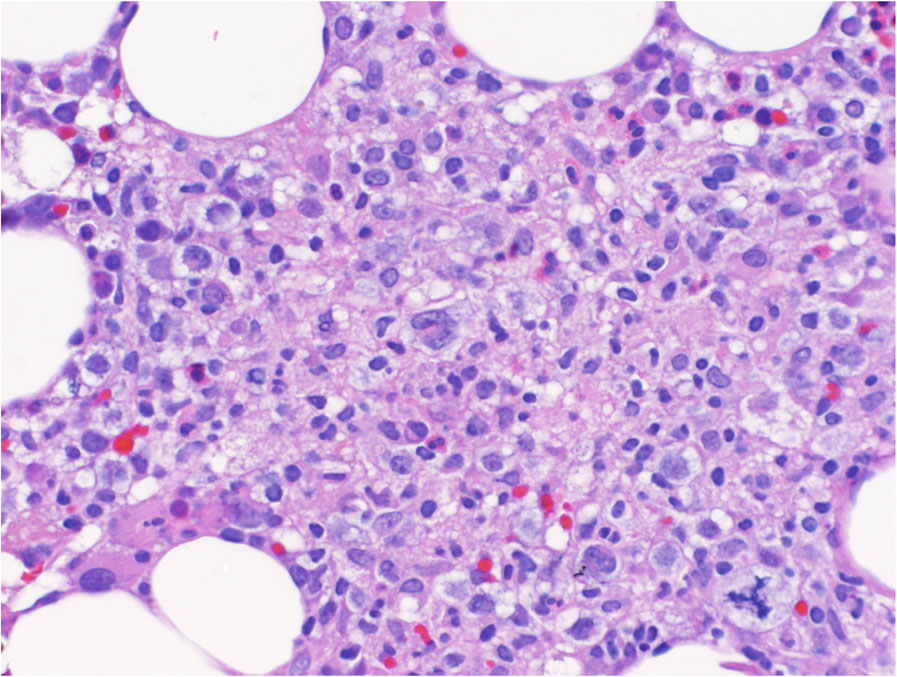
The bone marrow clot section showed focal areas contained rare large atypical cells (*center of image*) in a mixed background consisting of granulomatous inflammation, lymphocytes, eosinophils, and plasma cells. An atypical mitotic figure is present in the lower right corner. Hematoxylin and eosin, 400×

**Fig. 3:**
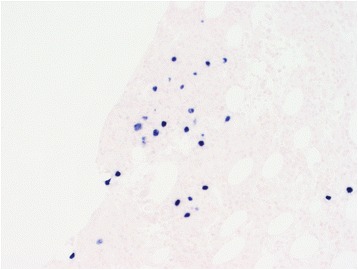
Areas with granulomatous inflammation in the bone marrow were positive for Epstein–Barr virus-encoded small RNA by *in situ* hybridization (200×)

On day 7, a bone marrow biopsy was performed which revealed a normocellular bone marrow (50%) with increased histiocytes with areas of hemophagocytic activity (Fig. 1). Focally, there were ill-defined granulomas present that contained few large atypical cells (Fig. 2). Paraffin immunohistochemistry showed positivity for small and large cells with CD30 and EBV-encoded small RNA (EBER), as well as few large cells with MUM-1 (Fig. 3). These findings were most compatible with atypical immunoblasts in the setting of an EBV infection, but this would be unusual in an immunocompetent adult. An additional consideration was the possibility of subtle involvement by Hodgkin lymphoma. The report suggested further investigation into a possible occult lymphoma.

On day 10, he was pre-emptively started on treatment for HLH per the HLH-94 treatment protocol with etoposide (initially at 75% dose reduction due to hepatic impairment, subsequent graduated increases in dose by 25%) and dexamethasone at 10 mg/m^2^ daily. This was continued for a 2-week course. Further laboratory testing revealed that he met five out of eight criteria for HLH diagnosis: fevers, pancytopenia, hemophagocytosis, ferritin of 23,292 ng/mL (>500 ng/mL), and soluble-CD25 of 15,330 pg/mL (>1033 pg/mL). He did not meet criteria for hypertriglyceridemia (161 mg/dL, less than 265) and hypofibrinogenemia (368 mg/dL, more than 150 mg/dL); natural killer (NK) cell activity was not tested. A subsequent liver biopsy on day 13 revealed findings of chronic hepatitis and focal histiocytic proliferation consistent with HLH.

**Fig. 4:**
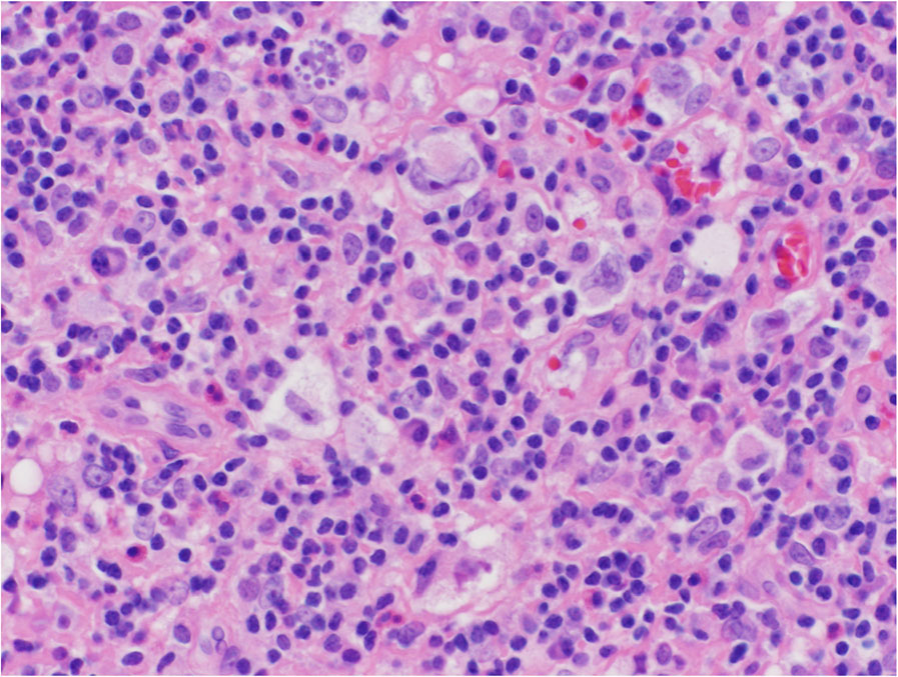
The subsequent cervical lymph node biopsy shows large Reed–Sternberg cells and variants in a background of mixed inflammation, diagnostic of classical Hodgkin lymphoma. Hematoxylin and eosin, 400×

With still no clear identifying etiology for secondary HLH, underlying malignancy was suspected and a positron emission tomography (PET) scan was performed. This revealed an enlarged right cervical lymph node measuring up to 1.7 cm as well as increased metabolic activity noted in his ascending colon. On day 18, a lymph node biopsy was performed which histologically revealed CD15, CD30, MUM-1, and EBER-positive cells with morphologic findings of classical Hodgkin lymphoma, lymphocyte-rich subtype (Fig. 4). On day 25, after 2 weeks of HLH-directed therapy with etoposide and dexamethasone, he had minimal improvement in his pancytopenia and hepatic impairment. He was then started on R-ESHAP for concurrent treatment of HLH with underlying Hodgkin lymphoma. He completed one full cycle of R-ESHAP (rituximab 375 mg/m^2^ day 25, etoposide 40 mg/m^2^ days 26 to 29, methylprednisolone 500 mg days 26 to 28,cisplatin 25 mg/m^2^ days 26 to 29, and cytarabine dose reduced to 1000 mg/m^2^ day 30). Only cytarabine was 50% dose reduced based on hepatic function. Initially he showed improvement in serial hepatic function panels. His EBV viral load 4 days after rituximab therapy (day 29 from diagnosis) was below detectable limits. However, his treatment course in the setting of continued neutropenia was complicated by *Enterococcus faecalis* bacteremia and colonic microperforation. He was not deemed to be a surgical candidate and his condition did not improve on broad spectrum antibiotics. Subsequently, he was transitioned to comfort measures only and moved to an in-patient hospice and died.

## Discussion

HLH is primarily a pediatric syndrome and babies are most commonly affected [5]. This form, known as primary HLH, is usually secondary to genetic abnormalities with known mutations in perforin 1, *UNC13D*, syntaxin 11, syntaxin-binding protein-2, *SH2D1A*, *RAB27A*, and *XIAP* or a defect on chromosome 9q21.3-22 [6]. It is inherited in an autosomal recessive pattern in up to 25% of the familial cases [7].

Primary HLH is a syndrome of immune activation and Stepp *et al*. first demonstrated perforin mutations as plausible etiologies [8]. Further animal studies have shown increased activation of CD8^+^ T cells and increased interferon gamma production in mice with perforin mutations [9]. In a separate study, gene expression data suggest that there is a marked imbalance of upregulation of proinflammatory proteins (IL-8, IL-6, CCL3, and CCL4) and anti-inflammatory proteins (TGF-β, IL-1RA, TNFAIP3, and IL-10) [10].

Secondary HLH, on the other hand, is less age restricted and is more common in adults. This form of HLH can be associated with an underlying infection, malignancy, or autoimmune disorder [11–15]. Malignancies leading to secondary HLH may reflect a similar paradigm of increased inflammatory cytokines originating from malignant cells. In our case, the inflammatory process is assumed to be driven by Hodgkin lymphoma in addition to EBV-affected T cells. The prognosis for HLH is poor in general but patients with malignancy-associated HLH have a markedly worse survival [15]. A recent Swedish study demonstrated that all the patients with malignancy-related HLH had died, with a dismal median survival of 22 days [3]. The most commonly reported malignancies associated with secondary HLH are non-Hodgkin lymphoma, particularly T-cell, and NK cell lymphoma, which are both strongly associated with EBV positivity [16]. Classical Hodgkin lymphoma leading to HLH is relatively rare. A retrospective review of a multicenter study cited just 17 out of 162 cases of HLH secondary to Hodgkin lymphoma, and another review of a single-center study reported only 1 out of 62 cases [17, 18]. Of interest, the prevalence of EBV positivity in HLH has been reported as high as 94% in Hodgkin disease compared to the typical prevalence of 30% [19, 20].

Overall, timeliness is imperative in the management of HLH. Previous case series report that adults with HLH carry a 30-day mortality of up to 44% and an overall mortality of up to 75% [21]. In an attempt to secure earlier diagnosis, scoring systems such as the HScore have been used for adult populations [22]. The HScore offers an estimated numerical probability of secondary HLH through the use of a cumulative integer score derived from variables including ferritin level, ALT, and degree of cytopenia. The currently accepted diagnostic criteria from clinical trials HLH-94 and HLH-2004 are from the pediatric population. With HLH being more frequently identified in the adult population, the 2004 criteria must be modified to prevent missed diagnoses. A recent retrospective analysis at University of Texas MD Anderson Cancer Center analyzing cases of pathologically confirmed HLH suggested an 18-point HLH diagnostic criteria for malignancy-associated HLH [4]. Ongoing clinical trials are underway in continued efforts to try to achieve early diagnosis [23].

Treatment for HLH with underlying malignancy is two-pronged: immunosuppression targeting the overt inflammation and cytotoxic chemotherapy targeting the malignancy. Effective treatment of HLH in our patient raised significant challenges. The established treatment for Hodgkin lymphoma in the form of adriamycin, bleomycin, vinblastine, and dexamethasone (ABVD) chemotherapy is very effective and has been used for decades with response rates in excess of 70% even in patients with poor prognosis [24]. However, in the case of our patient with underlying HLH and a total bilirubin >5 mg/dL, the treatment options are limited. Cytotoxic chemotherapies such as adriamycin and vinblastine cannot be safely used with severely impaired liver function. In addition, our patient’s EBER positivity was also taken into consideration in the fabrication of a treatment regimen. These challenges in therapy are common in patients with HLH; it implies that patients often will need individualized therapies. Our selection of R-ESHAP therapy was made in an attempt to optimize safety and efficacy.

The role of rituximab in EBV-positive Hodgkin lymphoma has shown some early promise. A known phase II study has demonstrated a significant decrease in plasma EBV positivity, and there is ongoing interest in the incorporation of rituximab in patients with EBV-positive lymphomas leading to HLH [25]. Thus far, there are several case reports reporting the effectiveness of rituximab in EBV-related HLH proposing success through limitation of inflammatory response from infected B cells [26, 27]. Our patient’s treatment with rituximab showed a positive effect with a reduction in his viral load to undetectable ranges.

The selection of ESHAP was primarily due to its known efficacy as salvage therapy in patients with relapsed Hodgkin lymphoma. Promising results were shown in using ESHAP as a less toxic salvage chemotherapy regimen for Hodgkin lymphoma [28, 29]. An etoposide-based treatment for secondary HLH from underlying malignancy has been previously recommended to address the overactive immune system [21]. The application of ESHAP to our patient did show evidence of initial improvement in his liver disease, but he ultimately succumbed to complications from chemotherapy.

As for now, cytotoxic chemotherapy remains the mainstay of treatment for adults with HLH secondary to an underlying malignancy. However, this is often not well tolerated in such a severe state of inflammation and multi-organ failure. This case highlights a common difficulty in treating patients with malignancy-associated HLH: treating HLH versus treating the underlying malignancy. Due to the poor therapeutic response to HLH-directed therapy in our patient, and the revelation of an underlying diagnosis, a switch was made from HLH-directed therapy to Hodgkin lymphoma-targeted therapy. Subsequently, our patient developed severe chemotherapy-related complications. Future considerations could be made to target the EBV with weekly rituximab while continuing etoposide for HLH in order to investigate if EBV rather than Hodgkin lymphoma may be driving the inflammatory process.

Fortunately, there is a recent increase in prevalence of targeted immunotherapy in Hodgkin lymphoma. Brentuximab vedotin is an antibody-drug conjugate targeting CD30 and is currently US Food and Drug Administration (FDA) approved for patients with Hodgkin lymphoma who are post-autologous hematopoietic stem cell transplant with high risk for relapse [30]. The emergence of lenalidomide, an antimetabolite with immunomodulatory activity, and nivolumab, a programmed cell death protein 1 (PD-1) inhibitor, present as feasible options for relapsed and refractory Hodgkin lymphoma [31–33]. It may be beneficial to assess the roles of these drugs as a first-line agent in immunocompromised or older patients who are not suitable candidates for cytotoxic chemotherapy.

The emergence of new drugs may also introduce promising prospects in the treatment of HLH. Ruxolitinib, a Janus kinase 1/2 (JAK 1/2) inhibitor, was shown to have good *in vivo* activity in mouse models with HLH [34]. A pilot study with this drug in patients with HLH is already underway (NCT02400463). Other novel agents in clinical trials include tocilizumab, a monoclonal antibody against IL-6 receptor (NCT02007239), low-dose IL-2 (NCT02569463), and alemtuzumab, a monoclonal antibody against CD52 in combination with etoposide and dexamethasone (NCT02385110).

## Conclusions

Overall, treatment of HLH continues to be a formidable challenge. Rare presentations of the disorder such as underlying Hodgkin lymphoma significantly alter the clinical course and treatment options. Treatment with R-ESHAP may be a viable option for patients with secondary HLH and Hodgkin lymphoma who cannot tolerate standard therapies due to hepatic impairment. With improvement in management of both Hodgkin and non-Hodgkin lymphoma as well as the arrival of targeted agents there may be more viable options that can be used as first-line therapy. The results of new, early phase trials including ruxolitinib will hopefully improve survival for these patients in future.

## Abbreviations

ABVD: Adriamycin, bleomycin, vinblastine, dexamethasone
ALT: Alanine aminotransferase
AMA: Antimitochondrial antibody
ANA: Antinuclear antibody
ASMA: Anti-smooth muscle antibody
AST: Aspartate aminotransferase
CMV: Cytomegalovirus
EBER: Epstein–Barr virus-encoded small RNA
EBV: Epstein–Barr virus
FDA: Food and Drug Administration
HHV-8: Human herpes virus-8
HIV: Human immunodeficiency virus
HLH: Hemophagocytic lymphohistiocytosis
HSV: Herpes simplex virus
INR: International normalized ratio
JAK 1/2: Janus kinase 1/2
MCV: Mean corpuscular volume
MRCP: Magnetic resonance cholangiopancreatography
NK: Natural killer
PD-1: Programmed cell death protein 1
PET: Positron emission tomography
R-ESHAP: Rituximab, etoposide, methylprednisolone, high-dose cytarabine, and cisplatin
RF: Rheumatoid factor
RNA: Ribonucleic acid
